# Digital pathology in clinical consultation practice

**DOI:** 10.4103/2153-3539.68334

**Published:** 2010-08-10

**Authors:** Subodh M. Lele

**Affiliations:** Department of Pathology and Microbiology, University of Nebraska Medical Center, Omaha, NE, USA

## BACKGROUND

Digital pathology or whole slide imaging technology in clinical consultation practice to me translates into convenient, effective and efficient communication. A not so old method of communication that we still use today is the wired telephone. As we all know, it has limitations in its usage due to it being “wired” and therefore not convenient to use, say for example, while walking down the street. Search for a better device eventually led to the development of the cellular phone. This provides for the required convenience factor without compromising the other features of the standard telephone. Cellular phones have evolved quite dramatically over the last few years such that the most recent versions of the phone with their numerous applications have become almost indispensable for daily use for many. However, the full potential of this device has still not been realized, especially in the field of telemedicine and telepathology. With the development of the new tablet computer/phone, one may not only read the newspaper but could also view whole scanned slides. Soon, it may be possible to read whole slide scans and also sign them out as one would do in his/her office, essentially from anywhere at anytime, using such portable devices!

## INTRAMURAL CONSULTATION

When I think of consulting another pathologist in my group in daily practice, I think of getting up from my chair in my office and traversing the office maze to get to the consultant. Valuable professional time is lost for communicating the difficult case to the consultant. It is also very inconvenient for the consultant to be interrupted in his/her work. In the not too distant future, with whole slide imaging, it may be possible to simply drag the difficult case, or more appropriately the whole slide scan icon for the case, and drop it into the consultant’s folder. The consultant would find it very efficient to simply open the folder and view the case(s) at his/her convenience at pretty much any time and from anywhere, even from bed after a hard day at the practice! This technology would also be especially useful when many pathologists from a group are away, for example, for attending a pathology meeting. Their expertise could still be utilized without any compromise in turn-around-time (TAT) or quality of care.

## GLOBAL CONSULTATION

I, not unlike many other pathologists, suffer from certain inertia when I think of sending a case for an outside consultation. The paperwork, recuts and the delay in getting the case across are the major factors. However, now with whole slide imaging and the appropriate software, with as few as two mouse clicks one may be able to have the scan(s)/case reviewed by essentially any pathologist from anywhere in the world.

## WHO CAN BENEFIT?

Essentially, any pathologist in any type of practice can benefit form this. The examples for this are given below.

1. *Pathologist in solo practice*: Of all the types of practices, this is the most obvious choice that would benefit from whole slide imaging technology. In addition to difficult cases or difficult intraoperative frozen sections, second signatures for malignant diagnoses, guidance on working up a case and quality assurance are some of the scenarios where digital pathology would benefit the pathologist in a solo practice.
2. *Pathologists in a small group practice*: Cases which would need subspecialty expertise that is lacking among the pathologists in that group are an ideal choice for an outside digital consultation. However, other examples include consulting other pathologists from within the group when many are away at national meetings.
3. *Pathologists with limited access to scientific material*: Pathologists in small practices may not have the accessibility to download scientific articles with the ease that is taken for granted in large academic institutions. Cases that may need more current data could easily be transmitted to the appropriate consultant who has the required access to data.
4. *Pathologists in large academic centers*: Uncommon/rare cases may need the opinion of an outside expert. The rapid transmission and access of slides in a digital format helps to avoid a compromise in TAT. Subspecialty expertise from within the group can also be relied upon at all times, given the ease of accessing digitized cases.

## TYPES OF CONSULTS

When one thinks of consultation in anatomic pathology, difficult/rare/challenging cases or difficult frozen sections come to our mind. These certainly would benefit from whole slide imaging; however, there are many other instances for which digital imaging technology would be appropriate. These include:

1. *Second signature for malignancies*: This would apply especially to those in solo or small group practices.
2. *Assistance in working up a case*: This again would be particularly useful to a pathologist in a solo or small group practice.
3. *Boost the confidence of a new pathologist*: A pathologist, fresh out of training, may need more frequent consultation. By simply dragging the icon for a case(s) into an appropriately titled folder, any pathologist from within the group or a designated pathologist from the outside could view the cases and render an opinion on them.
4. *Interpret/develop a special stain*: If a special stain is not working out the way it has been described in a scientific journal, the slides (with various dilutions and antigen retrieval methods) could be scanned and sent to the author or any outside consultant for advise on interpretation and troubleshooting.
5. *Consensus conferencing*: Virtual daily/weekly consensus conferencing on interesting/difficult cases within a pathology group can be arranged without anyone leaving his/her office/gross room. Conferences for consensus can also be similarly arranged with other pathology groups located anywhere in the world.
6. *Quality assuranc*e: Any remote pathology group can be contracted for quality assurance review of cases using the digital format.

## HOW DO YOU CONSULT?

The methods for enabling global consultation in digital format involve one or more of the following:

1. Uploading the case/digitized slides to a website on the Internet that can be accessed by another pathologist.
2. Software that enables you to share your desktop with an outside consultant. The consultant can then open and view the case(s)/slide(s) on your desktop and render his/her opinion.

PathXchange© is an example of a website on the Internet that allows for uploading of digitized slides. The following are some of its features:

1. No cost for uploading/sharing cases/forming groups;
2. Anyone can be a member, form a group and invite members to share cases;
3. Access to any group can be preset by its members;
4. A “write in” box appended to each case enables anyone to comment on the case;
5. The slide label is deleted when uploading a slide maintaining confidentiality;
6. No live conferencing is required, which helps in viewing cases at one’s own convenience and can be combined with any other software that allows for sharing one’s desktop to enable live conferencing.

WebEx© is an example of an application that allows for live web conferencing and sharing of one’s desktop. The following are some of its features:

1. Involves a cost for having a meeting;
2. Allows for live conferencing;
3. A viewing box enables participants to see each other or also to view gross specimens “live”;
4. Enables live sharing of one’s desktop with other participants and
5. Voice communication is via telephone and involves dial up access to start the meeting.

### Factors to Consider in Global Consultation of Digitized Cases

1. Security of the system and protection of patient information.
2. Other regulatory and visual interpretative issues that pertain to digital pathology, in general, as compared to glass slide reads. These include licensing, scan speed, image resolution and monitor display colors, to name a few.

## CONCLUSION

**Figure 1:**
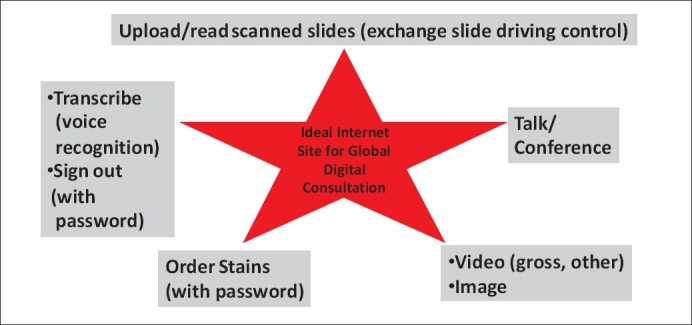
Features of an ideal system for global clinical consultation

Digital pathology provides quick access to cases, essentially from anywhere in the world and at any time. The ease with which one can view digitized slides provides many scenarios for its applicability in routine clinical pathology practice. At present, there are only a few systems available to share digitized slides with any pathologist from anywhere, some of which can be combined with live conferencing. In the future, more systems will likely become available with multitasking capabilities as shown in Figure 1. System security and regulatory issues (e.g. licensing, billing) would need to be examined as global digital consultation becomes more popular.

